# Anti‐Inflammatory Effects of Quercetin on High‐Glucose and Pro‐Inflammatory Cytokine Challenged Vascular Endothelial Cell Metabolism

**DOI:** 10.1002/mnfr.202000777

**Published:** 2021-01-22

**Authors:** Besim Ozyel, Gwénaëlle Le Gall, Paul W. Needs, Paul A. Kroon

**Affiliations:** ^1^ Nutrition and Dietetics Department European University of Lefke Lefke, Northern Cyprus, TR‐10 Mersin 9910 Turkey; ^2^ Norwich Medical School University of East Anglia Bob Champion Research and Education Building, James Watson Road, Norwich Research Park Norwich NR4 7UQ UK; ^3^ Quadram Institute Bioscience Norwich Research Park Norwich NR4 7UQ UK

**Keywords:** glycemia, inflammation, metabolome, polyphenols, purine metabolism

## Abstract

**Scope:**

Pro‐inflammatory stimuli such as hyperglycemia and cytokines have been shown to negatively affect endothelial cell functions. The aim of this study is to assess the potential of quercetin and its human metabolites to overcome the deleterious effects of hyperglycemic or inflammatory conditions on the vascular endothelium by modulating endothelial cell metabolism.

**Methods and results:**

A metabolomics approach enabled identification and quantification of 27 human umbilical vein endothelial cell (HUVEC) metabolites. Treatment of HUVECs with high‐glucose concentrations causes significant increases in lactate and glutamate concentrations. Quercetin inhibits glucose‐induced increases in lactate and adenosine 5′‐triphosphate (ATP) and also increased inosine concentrations. Tumor necrosis factor α‐treatment (TNFα) of HUVECs causes increases in asparagine and decreases in aspartate concentrations. Co‐treatment with quercetin reduces pyruvate concentrations compared to TNFα‐only treated controls. Subsequently, it was shown that quercetin and its HUVEC phase‐2 conjugates inhibit adenosine deaminase, xanthine oxidase and 5′nucleotidase (CD73) but not ectonucleoside triphosphate diphosphohydrolase‐1 (CD39) or purine nucleoside phosphorylase activities.

**Conclusion:**

Quercetin was shown to alter the balance of HUVEC metabolites towards a less inflamed phenotype, both alone and in the presence of pro‐inflammatory stimuli. These changes are consistent with the inhibition of particular enzymes involved in purine metabolism by quercetin and its HUVEC metabolites.

## Graphical abstract

Pro‐inflammatory stimuli such as hyperglycemia and cytokines have been shown to negatively affect endothelial cell functions. In the present study, quercetin is shown to alter the balance of HUVEC metabolites towards a less inflamed phenotype, both alone and in the presence of pro‐inflammatory stimuli. These changes are consistent with inhibition of particular enzymes involved in purine metabolism by quercetin and its HUVEC metabolites.

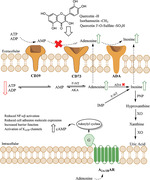

## 1 Introduction

According to World Health Organization, cardiovascular disease (CVD) is the leading cause of death globally.^[^ ^1^ ^]^ The major contributor to CVD is atherosclerosis, and it is characterized as a chronic inflammatory disease.^[^ ^2^ ^]^ Diabetes is known to accelerate atherosclerosis however, the mechanisms are not fully elucidated.^[^ ^3^ ^]^

Endothelial cells are responsible for various important processes such as regulating vessel permeability, inflammation, and thrombosis.^[^ ^4^ ^]^ Endothelial cell dysfunction which leads to decreased bioavailability of nitric oxide has an impact on endothelial cell activation where enhanced cell permeability, elevated cell adhesion molecule expression and reduced anti‐thrombotic properties are observed.^[^ ^5^ ^]^ Therefore, endothelial cell dysfunction and endothelial cell activation concomitantly disrupt endothelial cell function initiating atherosclerotic plaque formation.^[^ ^6^ ^]^

High‐glucose concentrations experienced during diabetes generate inflammatory stimuli, which lead to endothelial cell dysfunction and endothelial cell activation. Reduced nitric oxide production,^[^ ^7^ ^]^ increased nuclear factor‐kappaB (NF‐кB) activation,^[^ ^8^ ^]^ increased cell adhesion molecule expression^[^ ^9^ ^]^ and expression of inflammatory genes^[^ ^10^ ^]^ have been reported when endothelial cells were exposed to hyperglycemic environment. There are also numerous reports providing evidence of the anti‐inflammatory^[^ ^11^ ^]^ and anti‐apoptotic^[^ ^12^ ^]^ effects of polyphenols when endothelial cells have been exposed to hyperglycemic and pro‐inflammatory conditions. To date, we are only aware of one such report concerned with the effects of quercetin metabolites on high‐glucose treated vascular endothelial cells.^[^ ^13^ ^]^ These previous reports focused on targeted effects, particularly inflammation responses and changes in gene expression and signaling. Non‐targeted approaches are useful for identifying a broad range of effects and potentially unanticipated effects of changes in conditions (e.g., glycemia, inflammation) on cell functions. Evidence of links between changes in inflammation and cell metabolism have emerged^[^ ^14^, ^15^ ^]^ and this directed us to use non‐targeted metabolomics approach to investigate the effects of high glucose and inflammatory cytokine treatments on cellular metabolism.

Cultured endothelial cells are used to represent the vascular endothelium. However, they are not homogenous which means that endothelial cells from different vascular beds may behave differently in response to various stimuli.^[^ ^16^ ^]^ A previous study revealed the heterogeneity of responses to pro‐inflammatory cytokines TNFα, IL‐1β, and LPS by selected arterial and venous endothelial cells where human umbilical vein endothelial cells (HUVECs) developed comparatively strong inflammatory responses reflected as increased cell adhesion molecule expression, increased monocyte adhesion, and phosphorylation of signaling molecules such as extracellular signal‐regulated protein kinases 1 and 2 involved in inflammation.^[^ ^17^ ^]^ In another study, glucose transporters in arterial, venous, and capillary endothelial cells were examined showing that HUVECs have similar glucose transporter mRNA expression pattern and also comparable protein levels of most abundantly found transporters, glucose transporter 1 and 3, to these particular endothelial cells.^[^ ^18^ ^]^ Consequently, a HUVEC model which has been extensively used in diabetes and CVD research was employed in this study.^[^ ^19^ ^]^

The aim of the present study was to evaluate the ability of quercetin and its phase‐2 conjugates to overcome the pro‐inflammatory effects of hyperglycemia and cytokine treatments in HUVECs. The data showed that quercetin and its conjugates alter HUVEC energy metabolism, and we went on to provide evidence that the observed changes in HUVEC energy metabolism may be a consequence of direct interaction of quercetin and its conjugates with the enzymes involved in purine metabolism.

## 2 Experimental Section

### 2.1 Cell Culture

HUVECs were obtained from Cambrex Bio Science (Wokingham, UK), and grown in endothelial cell basal growth medium (EBM‐2, 5.5 mm glucose) supplemented with fetal bovine serum, nutrients, and growth factors (EGM‐2 SingleQuots). HUVEC cultures were maintained at 37 °C in an atmosphere at 5% CO_2_.

### 2.2 High‐Glucose, TNFα, and Polyphenol Challenge of HUVECs

After HUVECs reached confluence, culture medium was replaced with high‐glucose medium (28.5 mm) for 18 h or medium containing TNFα (10 ng mL^−1^) for 6 h. Polyphenol treatments involved additional 2 h pre‐treatment with quercetin (10 μm) followed by 18 h of high‐glucose (28.5 mm) or 6 h of TNFα (10 ng mL^−1^) co‐treatment.

### 2.3 Cell Quenching and Metabolite Extraction

First, a protocol that allowed quick arrest of cellular metabolism and efficient extraction of metabolites using existing literature methods as a starting point was optimized.^[^ ^20^, ^21^ ^]^ 80% HPLC grade methanol (−80 °C, 3 ml) was used to quench the cells. After quick removal of culture medium from the culture dish (10 cm^2^), cold methanol was added. This was followed by 15 min incubation on dry ice. A cell‐scraper was used to detach the cells and disrupt the cell membranes. The methanol solution containing HUVEC metabolites was collected into a 5 mL centrifuge tube and centrifuged (2000 g/ 5 min at 4 °C). The supernatant was saved and pellet was re‐constituted in 0.5 ml of 80% HPLC grade methanol. Centrifugation step was repeated. The supernatants were pooled and dried using a centrifugal evaporator.

### 2.4 Sample Preparation and NMR Acquisition

Dried intracellular extracts were re‐constituted in a known amount of buffer containing Na_2_HPO_4_, NaH_2_PO_4_, NaN_3_ and TSP (sodium 3‐(trimethylsilyl)‐propionated_4_) in D_2_O. A 600 MHz Bruker Avance spectrometer fitted with a 5 mm TCI cryoprobe and a 60 slot auto‐sampler (Bruker, Germany) was used to acquire high‐resolution ^1^H NMR spectra with a 1D NOESY pulse sequence (NOESYPR1D). Samples were held at a constant temperature of 26.85 °C. 2.05s acquisition involved 128 scans of 32768 complex data points, and the spectral width was 13.3 ppm. A low power selective irradiation was applied at the water frequency to suppress the residual water signal during the recycle delay (D1 = 2s) and mixing time (D8 = 0.10s). All spectra were phase‐ and baseline‐corrected manually using the TOPSPIN 2.0 software.

### 2.5 Hydrophilic Interaction Chromatography (HILIC) Mode LC‐MS/MS Analysis

Levels of adenosine 5′‐triphosphate (ATP), adenosine 5′‐ diphosphate (ADP), adenosine 5′‐ monophosphate (AMP), adenosine, inosine, and xanthine were measured in both intra‐ and extracellular samples. Prior to MS analysis, dried extracts were dissolved in H_2_O. An Agilent 1200 HPLC system (Waldbronn, Germany) coupled with an Applied Biosystems 4000 QTrap triple quadrupole MS was employed using the analytical method described by Preinerstorfer et al. (2010).^[^ ^22^ ^]^ Analyst 1.5 software (Applied Biosystems) was used to process data.

### 2.6 LC‐DAD and LC‐MS Analyses

Media samples were mixed using a bench top vortex, and centrifuged at 13 000 rpm for 10 min at 4 °C using a bench top centrifuge. Intracellular extracts were mixed using a bench top vortex, and ultra‐sonicated in a sonic water bath for 10 min and then centrifuged at 13.000 rpm for 10 min at 4 °C. Supernatants were analyzed with either LC‐DAD or LC‐MS.

Reverse phase HPLC analyses were conducted using an Agilent HP1100 system (Waldbronn) with ultraviolet diode array detection. During chromatographic runs, autosampler temperature was set to 4 °C, temperature of the column compartment was 30 °C and injection volumes were between 20 and 100 μL. Metabolites were separated using a Phenomenex Luna 5‐C18(2) (250 × 4.60 mm, 5 μm) column at a flow rate of 1 mL min^−1^. Mobile phases were prepared as A) 50 mm ammonium acetate in H_2_O (adjusted to pH 5) and B) 2% THF and 0.1% acetic acid in acetonitrile. Gradient elution was detailed in the Supporting Information.

An Agilent 1100 HPLC system coupled with an Agilent LC/MSD SL spectrometer was used for LC‐MS analyses. The same gradient elution was used for the LC‐MS analyses. MS in full scan in both positive and negative ion modes with electrospray ionization was used to detect quercetin conjugates. Individual standards for quercetin, isorhamnetin, rhamnetin, tamarixetin, quercetin 3′‐O‐sulfate (Q3′‐O‐S), quercetin 3‐O‐sulfate (Q3‐O‐S), quercetin 3′‐glucuronide (Q3′‐O‐GlcA), quercetin 3‐glucuronide (Q3‐O‐GlcA), and isorhamnetin 3‐glucuronide (IsoR3‐O‐GlcA) were analyzed using LC‐DAD and LC‐MS methods in order to help the metabolite identification process and for quantification.

### 2.7 Enzymatic Assays

#### 2.7.1 Adenosine Deaminase and Xanthine Oxidase

The effects of quercetin and its conjugates on adenosine deaminase (ADA) (recombinant human adenosine deaminase expressed in *Escherichia coli*) and xanthine oxidase (XO) (from bovine milk) activity were tested using commercial enzymes (Sigma‐Aldrich) and the continuous spectrophotometric rate determination assay procedures provided by the supplier.

Furthermore, ADA activity in HUVEC protein extracts was tested. First, the confluent HUVECs were washed three‐times with PBS (room temperature) followed by the incubation on dry ice for 15 min. Lysate was obtained by scraping the cells using a cell scraper after addition of 400 μL pure water and transferred into an Eppendorf tube. The solution was centrifuged at 4 °C for 15 min. Commercial enzyme was replaced with an extract containing 30 μg protein in the assay.

#### 2.7.2 Purine Nucleoside Phosphorylase

The effects of quercetin and its metabolites on purine nucleoside phosphorylase (PNP) (*Geobacterium* sp. PNP expressed in *Escherichia coli*) were tested using both commercial enzyme (Sigma‐Aldrich) and HUVEC extracts using the continuous spectrophotometric rate determination procedure. A guanosine analogue, 7‐methyl‐6‐thioguanosine (MESG, Carbosynth Ltd), was used as the substrate for PNP. Assay buffer containing 50 mm potassium phosphate (pH 7.4) was prepared. It was used to dilute PNP to 0.2 ng μL^−1^ and substrate to 800 μm. 50 μL of substrate solution was added to each well and incubated for 1 min at 37 °C. Reaction was initiated by the addition of 50 μL of enzyme solution. The increase in absorption at 360 nm was recorded at 30 s intervals for 5 min at 37 °C measuring the formation of the product. PNP activity was also tested using HUVEC protein extracts. Procedure involved replacing commercial enzyme in the assay with HUVEC protein extract containing 30 μg protein.

#### 2.7.3 CD39 and CD73

The effects of quercetin and its metabolites on recombinant human (rh) ectonucleoside triphosphate diphosphohydrolase‐1 (NTPDase, CD39) and 5′nucleotidase (5′‐NT, CD73) (R&D Systems) activities were tested using a colorimetric phosphate quantification method (Biomol Green Reagent, Enzo Life Sciences) following the supplier's assay procedure.

An assay was adapted and optimized from previous studies^[^ ^23^, ^24^ ^]^ for testing the effects of quercetin and its conjugates on CD39 and CD73 in intact HUVECs. The first step in the assay involved removing cell culture medium and washing the cells grown to confluence in 6‐well plates three times with the assay buffer (25 mm Tris, 5 mm CaCl_2_ for CD39 or 5 mm MgCl_2_ for CD73, pH 8). This was followed by the incubation of cells with 2 mL of substrate buffer (assay buffer, 5 mm tetramisole HCl, 100 μm ATP for CD39 or 50 mm AMP for CD73, pH 8) at 37 °C for 15 min. Ecto‐alkaline phosphatase activity was inhibited with the addition of tetramisole HCl. Biomol Green reagent was used to quantify free phosphate levels. Inhibition studies involved addition of various concentrations of quercetin and its conjugates into substrate buffer. Insoluble quercetin conjugates were dissolved in DMSO (final concentration, 0.1% v/v) prior to their addition into substrate buffer. Two different sets of blanks were prepared. The first set contained substrate buffer without the substrate and quercetin/quercetin conjugates. These were incubated with the cells. The other set of blanks contained the substrate buffer containing the substrate without the cells.

### 2.8 Statistical Analysis

Amix software package (Bruker, Germany) was used to bucket and integrate water‐suppressed NMR spectra. These buckets covered 0.1–8.9 ppm (4.60–5.0 ppm was omitted due to water signals). The data matrix of the integrals of these buckets was used for principal component analysis (PCA) in the multivariate analysis that was performed using Matlab. Data were scaled to unit variance to compensate for large differences in intensity among metabolite signals. Buckets defined in a NMR spectrum represent a single data point in PCA, and they are responsible for the separation observed in score and loading plots.

Univariate statistical analyses were performed using Graphpad Prism 5.01 software. Non‐parametric Mann–Whitney *U* test was used to test significant differences between individual metabolite concentrations. *p* values less than 0.05 (*p < 0.05, **p < 0.01, ***p<0.001) were accepted as a significant difference.

## 3 Results

### 3.1 Effects of High‐Glucose and Quercetin on HUVEC Metabolites

Of the six different sample preparation methods tested, the optimized direct methanol extraction protocol was found to be the best performing in terms of quenching metabolism and extracting metabolites from cells. Extracted cellular metabolites were subjected to ^1^H NMR analysis which facilitated the identification of 27 intracellular metabolites. Details of the metabolites can be found in the Supporting Information.

**Figure 1:**
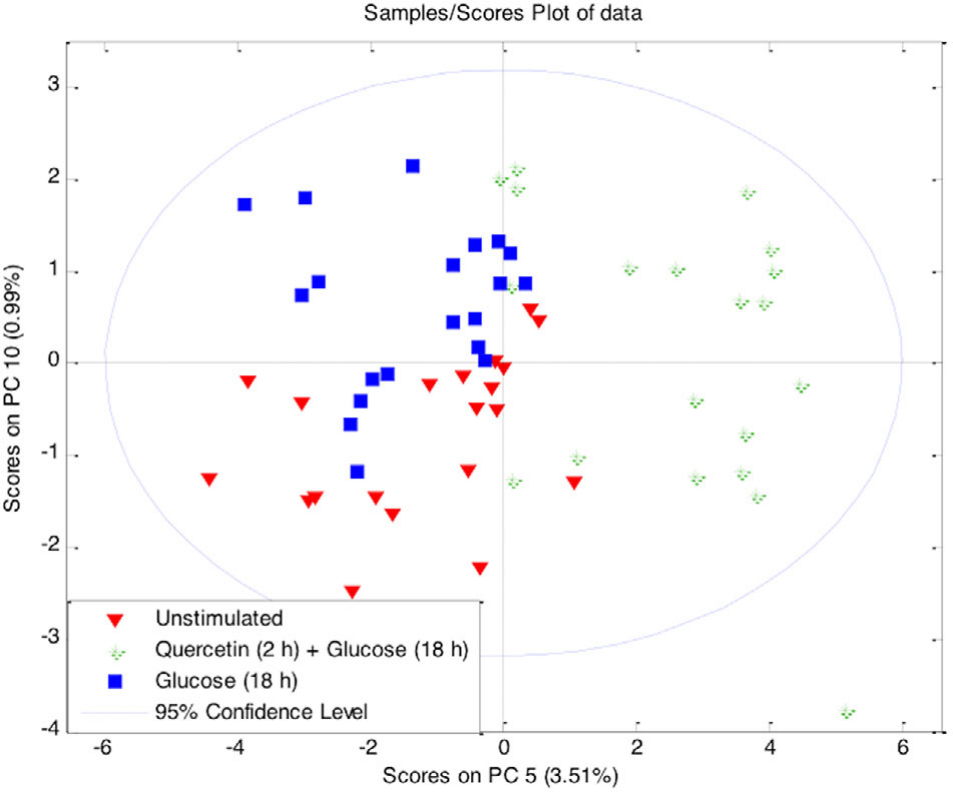
Multivariate analysis of altered HUVEC metabolic profile. PCA score plots of ^1^H NMR data showing separation of normal glucose (5.5 mm glucose for 18 h), high‐glucose (28.5 mm glucose for 18 h), and quercetin/glucose treated (10 μm quercetin for 2 h followed by 28.5 mm glucose for 18 h) HUVEC samples (*n* = 20).

There were three different treatment groups in the experimental design; normal glucose (5.5 mm glucose for 18 h), high‐glucose treated (28.5 mm glucose for 18 h), and quercetin/glucose treated HUVECs (10 μm quercetin for 2 h followed by 28.5 mm glucose for 18 h). A multivariate analysis showed that the groups were separated on the scores plot of data indicating alterations in HUVEC metabolic profiles with different treatments (**Figure** **1**). The metabolites that contributed significantly to this separation were identified as lactate, inosine, ATP, pyruvate, acetate, glutamate, aspartate, nicotinamide dinucleotide (NAD^+^) and asparagine after loading plot analysis. Furthermore, our univariate analysis (Mann–Whitney *U* test) confirmed the significant changes (**Table** **1**).

**Table 1:** Univariate analysis (Mann–Whitney *U* test) confirmed statistically significant alterations in the levels of metabolites which contributed to the separation in multivariate analysis. Metabolite levels in normal glucose (5.5 mm glucose for 18 h), high‐glucose (28.5 mm glucose for 18 h) and quercetin/glucose (10 μm quercetin for 2 h followed by 28.5 mm glucose for 18 h) treated HUVECs were compared. ↑, increase; ↓, decrease

Normal glucose (18 h) vs High glucose (18 h), *n* = 38			High glucose (18 h) vs Quercetin (2 h) /High glucose (18 h), *n* = 20		
Lactate	↑	*p* < 0.01	Inosine	↑	*p* < 0.001
Glutamate	↑	*p* < 0.05	Acetate	↑	*p* < 0.05
—		—	Lactate	↓	*p* < 0.01
—		—	ATP	↓	*p* < 0.01
—		—	NAD^+^	↓	*p* < 0.01
—		—	Pyruvate	↓	*p* > 0.05

### 3.2 Effects of TNFα and Quercetin on HUVEC Metabolites

**Figure 2:**
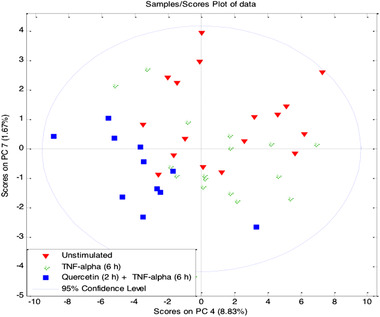
Multivariate analysis of altered HUVEC metabolic profile. PCA score plots of ^1^H NMR data showing separation of unstimulated (*n* = 17), TNFα treated (10 ng mL^−1^ TNFα for 6 h, *n* = 17), and quercetin/ TNFα treated (10 μm quercetin for 2 h followed by 10 ng mL^−1^ TNFα for 6 h, *n* = 11) HUVEC samples.

There were three different treatment groups in the experimental design; unstimulated (for 6 h), TNFα treated cells (10 ng mL^−1^ TNFα for 6 h), and quercetin /TNFα treated (10 μm quercetin for 2 h followed by10 ng mL^−1^ TNFα for 6 h) HUVECs. A multivariate analysis showed that the groups are separated on the scores plot of data indicating alterations in HUVEC metabolic profiles with different treatments (**Figure** **2**). The metabolites, which contributed significantly to this separation, were identified as pyruvate, NAD^+^, ATP, inosine, lactate, aspartate, asparagine, and histidine after loading plot analysis. Furthermore, our univariate analysis (Mann–Whitney test) confirmed the significant changes (**Table** **2**).

**Table 2:** Univariate analysis (Mann–Whitney *U* test) confirmed statistically significant alterations in the levels of metabolites which contributed to the separation in multivariate analysis. Metabolite levels in unstimulated, TNFα treated (10 ng mL^−1^ TNFα for 6 h) and quercetin/ TNFα (10 μm quercetin for 2 h followed by 10 ng mL^−1^ TNFα for 6 h) treated HUVECs were compared. ↑, increase; ↓, decrease

Unstimulated (6 h) vs TNFα (6 h), *n* = 17			TNFα (6 h) vs Quercetin (2 h) / TNFα (6 h), *n* = 11		
Asparagine	↑	*p* < 0.05	Inosine	↑	*p* = 0.056
Pyroglutamate	↑	*p* > 0.05	Pyruvate	↓	*p* < 0.05
Aspartate	↓	*p* < 0.001	Aspartate	↓	*p* = 0.057
—		—	ATP	↓	*p* > 0.05

### 3.3 LC‐MS Analysis of Metabolites Involved in Cellular Energy Metabolism

Having observed that the main treatment‐induced alterations in metabolites occurred in cellular energy metabolism, we employed what should have been a more sensitive method to quantify both intra‐ and extra‐cellular concentrations of the highly polar energy metabolites adenosine, inosine, xanthine, AMP, ADP, ATP, and NAD^+^ which would show poor retention on reversed phase columns. However, AMP, ADP, ATP, and NAD^+^ could not be detected in extracellular medium using this method.

High‐glucose treatment (28.5 mm, 18 h, *n* = 6) did not change intracellular concentrations of any of the metabolites compared to unstimulated cells (5.5 mm, 18 h, *n* = 6). However, subsequent quercetin treatment caused significant changes in several metabolites that are shown in **Table** **3** together with the changes in metabolites observed after TNF‐α stimulation and quercetin treatment. Among extracellular metabolites, inosine was the only one with a significant elevation (*p *< 0.01).

**Table 3:** LC‐MS analysis of changes in HUVEC energy metabolites. Changes in selected energy metabolites observed with quercetin pre‐treatment before high‐glucose (28.5 mm glucose for 18 h) and TNFα (10 ng mL^−1^ for 6 h) treatments. ↑, increase; ↓, decrease

High glucose (18 h) vs Quercetin (2 h)/High glucose (18 h), *n* = 6				TNFα (6 h) vs Quercetin (2 h)/TNFα (6 h), *n* = 6			
Intracellular		Extracellular		Intracellular		Extracellular	
Inosine*	↑	Inosine**	↑	Inosine***	↑	Inosine**	↑
ATP**	↓	Xanthine*	↓	Adenosine*	↑	—	
ADP*	↓	—	—	ATP*	↓	—	
NAD^+^ **	↓	—	—	—	—	—	

* *p* < 0.05

** *p* < 0.01

*** *p* < 0.001

Furthermore, time‐dependent effects of quercetin treatment on these metabolites were tested. Confluent HUVECs were incubated with 10 μm quercetin for 2, 8, or 20 h, and the changes in metabolites are shown in **Table** **4**. Among the metabolites quantified, xanthine was the only intracellular metabolite that was not affected by the treatments. When the extracellular media were analyzed, a significant elevation in adenosine concentrations (*p *< 0.01) was observed with 2 h quercetin treatments. In parallel, a time‐dependent elevation was observed in inosine concentrations. Xanthine concentration was also significantly reduced with 20 h quercetin treatment (*p* < 0.01).

**Table 4:** Alterations in metabolites involved in cellular energy metabolism after 2, 8, and 20 h quercetin (10 μm) challenges (*n* = 6). Metabolite levels of unstimulated cells compared to metabolite levels of quercetin treated cells at each time point. Data are presented as the mean ± standard deviation

	2 h	8 h	20 h	2 h	8 h	20 h
	Intracellular [%]			Extracellular [%]		
ATP	180 ± 90	56 ± 16**	71 ± 14**	—	—	—
ADP	—	—	72 ± 21*	—	—	—
AMP	97 ± 14	154 ± 40*	117 ± 29	—	—	—
Adenosine	286 ± 19*	73 ± 30	162 ± 65*	113 ± 6**	106 ± 16	103 ± 16
Inosine	122 ± 22	201 ± 41**	273 ± 35**	120 ± 3**	168 ± 4**	303 ± 13**
Xanthine	100 ± 17	103 ± 20	110 ± 13	103 ± 5	104 ± 11	94 ± 1**
NAD^+^	—	91 ± 10	76 ± 3**	—	—	—

* *p* < 0.05.

** *p* < 0.01.

*** *p* < 0.001.

### 3.4 Fate of Quercetin in the HUVEC Model

**Figure 3:**
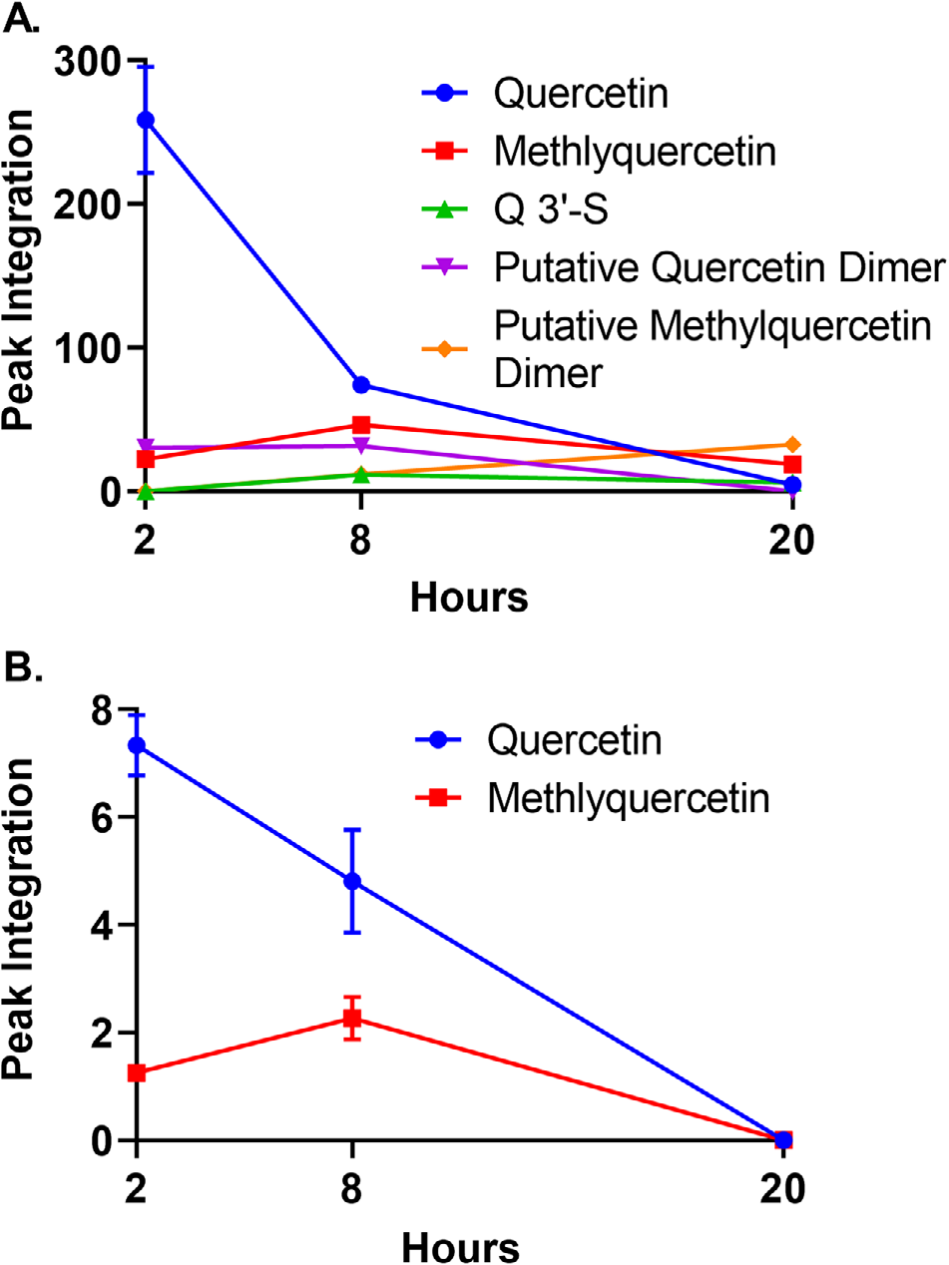
Uptake and metabolism of quercetin by HUVECs. A) Changes in quercetin and its conjugates in culture medium after incubating cells with 10 μm quercetin for 2, 8 and 20 h (*n* = 3). B) Changes in quercetin and its conjugates in intracellular fluids of HUVECs after incubating cells with 10 μm quercetin for 2, 8 and 20 h (*n* = 3). Data are presented as the mean ± standard deviation according to representative peak integrations of HPLC chromatograms for quercetin and quercetin conjugates.

Uptake kinetics of quercetin was determined by incubating the cells with 10 μm quercetin for 2, 8, and 20 h (**Figure** **3**). Quercetin was metabolized quickly into Q3′‐O‐S, methylquercetin and 2 other unidentified metabolites. Quercetin and methylquercetin was detected in intracellular extracts after 2 and 8 h. Q3′‐O‐S could not be detected intracellularly at any time point. However, it could be detected in culture media after 2 h treatment indicating that it was quickly metabolized and removed from the cells.

Samples containing quercetin, its conjugates and other two unidentified molecules were analyzed also using LC‐MS. The analysis confirmed the identities of the quercetin, Q3′‐O‐S and methylquercetin (details given in the Supporting Information). Since the retention times of isorhamnetin (3′‐OCH_3_‐quercetin) and tamarixetin (4′‐OCH_3_‐methylquercetin) were very similar, they could not be distinguished. Ultimately, the novel metabolites that did not correspond to any of our authentic standards could not be equivocally identified. However, LC‐MS analysis suggested that they are likely to be dimers of quercetin and methylquercetin.

### 3.5 Effects on Enzyme Activities

The inhibitory effects of selected flavonols (2 –70 μm) on activities of enzymes involved in purine metabolism were tested in both commercial enzymes and HUVEC extracts, and their IC_50_ (half maximal inhibitory concentration) values were calculated where it was possible (**Table** **5** and **Table** **6**).

**Table 5:** Effects of quercetin and its conjugates on ADA, XO, and PNP activities. Commercial enzymes and HUVEC extracts were used in enzyme assays to determine IC_50_ values (*n* = 3)

Enzyme	Inhibitor	IC_50_ [μM] for commercial enzyme	IC_50_ [μM] for HUVEC extract
Adenosine Deaminase	Isorhamnetin	48.9	15.7
	Quercetin	55.6	20.0
	Quercetin 3′‐O‐Sulfate	195	55.7
	Quercetin 3‐O‐Glucuronide	Not calculated	Not tested
Xanthine Oxidase	Isorhamnetin	0.31	Not tested
	Quercetin 3′‐O‐sulfate	0.45	Not tested
	Quercetin	3.0	Not tested
Purine Nucleoside Phosphorylase	Quercetin	36.9	No inhibition
	Quercetin 3′‐O‐sulfate	48.3	No inhibition
	Isorhamnetin	No inhibition	Not tested
	Quercetin 3‐O‐sulfate	No inhibition	Not tested
	Isorhamnetin 3‐O‐Glucuronide	No inhibition	Not tested
	Rhamnetin	No inhibition	Not tested
	Quercetin 3′‐O‐Glucuronide	No inhibition	Not tested
	Quercetin 3‐O‐Glucuronide	No inhibition	Not tested

**Table 6:** Effects of quercetin and its conjugates on enzyme CD39 and CD73 activities. Commercial enzymes and intact HUVECs were used in enzyme assays to determine IC_50_ values

Enzyme	Inhibitor	IC_50_ [μM] for commercial enzyme	IC_50_ [μM] for HUVEC extract
CD39	Quercetin 3′‐O‐sulfate	0.18	No inhibition
	Quercetin	0.57	No inhibition
	Quercetin 3′‐O‐Glucuronide	10.1	Not tested
	Quercetin 3‐O‐Glucuronide	52.5	Not tested
	Isorhamnetin 3‐O‐Glucuronide	71.2	Not tested
	Isorhamnetin	No inhibition	No inhibition
	Tamarixetin	No inhibition	Not tested
	Rhamnetin	No inhibition	Not tested
CD73	Quercetin	1.7	13% inhibition with 50 μm quercetin
	Isorhamnetin	8.3	No inhibition
	Quercetin 3′‐O‐sulfate	160	No inhibition
	Quercetin 3‐O‐Glucuronide	No inhibition	Not tested
	Isorhamnetin 3‐O‐Glucuronide	No inhibition	Not tested
	Quercetin 3′‐O‐Glucuronide	No inhibition	Not tested
	Rutin	No inhibition	Not tested

#### 3.5.1 Adenosine Deaminase

Isorhamnetin showed the most potent inhibitory activity which was followed by quercetin, Q3′‐O‐S and Q3‐O‐GlcA. Except Q3‐O‐GlcA, other conjugates tested showed dose‐dependent inhibitory effects. Nevertheless, Q3‐O‐GlcA inhibited ADA activity at 2 μm (*p *< 0.01, *n* = 3). The higher concentrations of Q3‐O‐GlcA did not affect the residual activity. This prevented us from calculating IC_50_ value for the Q3‐O‐GlcA. The inhibitory activities by quercetin and its conjugates were similar using both the commercial enzyme and the HUVEC extract. Also, it was shown that heating the HUVEC extracts to 70 °C halts the enzyme activity indicating that the loss of adenosine was enzyme dependent.

#### 3.5.2 Xanthine Oxidase

Similarly, isorhamnetin was the most potent inhibitor of xanthine oxidase (XO) activity followed by Q3′‐O‐S, and quercetin. Xanthine oxidase activity could not be detected in HUVEC extracts (30–100 μg protein content) although the extracts were de‐salted and concentrated using spin columns (Millipore Microcon) in order to prevent any substances interfering with the assay.

#### 3.5.3 Purine Nucleoside Phosphorylase

Bacterial purine nucleosidase activity was inhibited by only quercetin and Q3′‐O‐S with IC_50_ values of 36.9 and 48.3 μm (*n* = 3) respectively. The minimum concentrations of quercetin and Q3′‐O‐S to inhibit PNP activity significantly were 30 and 15 μm respectively. Phosphate and sulfate ions were also tested for their potential inhibitory activities alone or in conjunction with Q3′‐O‐S. Nevertheless, these ions did not cause any inhibition in enzyme activity by themselves or in conjunction with Q3′‐O‐S. After the observation of inhibitory activities of quercetin and Q3′‐O‐S, they were also tested for their inhibitory effects on PNP activity in HUVEC extracts. Nevertheless, quercetin aglycone and Q3′‐O‐S did not affect PNP activity in HUVEC protein extracts.

#### 3.5.4 CD39

The minimum concentrations of Q3′‐O‐S, quercetin and Q3′‐O‐GlcA to inhibit rhCD39 activity was 2 μm (*p* < 0.001). On the other hand, quercetin, IsoR3‐O‐GlcA, and Q3‐O‐GlcA required minimum 15 μm concentration to inhibit rhCD39 activity significantly (*p* < 0.05). Quercetin, Q3′‐S and isorhamnetin were tested and no effects were observed in the CD39 activity in intact HUVECs.

#### 3.5.5 CD73

The minimum concentrations of quercetin and isorhamnetin to inhibit rhCD39 activity significantly were 1 μm (*p* < 0.01) and 2 μm (*p* < 0.001) respectively. Only quercetin inhibited CD73 in intact HUVECs (50 μm, 13% inhibition, *n* = 3). Isorhamnetin and Q3′‐O‐S did not have any inhibitory effects.

## 4 Discussion

Under physiological conditions, endothelial cells are exposed to ≈5.5 mm glucose that represents the fasting blood glucose concentration. This value increases under conditions such as metabolic syndrome and diabetes in parallel with increases observed in pro‐inflammatory cytokine levels.^[^ ^25^, ^26^ ^]^ In our preliminary experiments, we have assessed the potential of glucose (10–35 mm), TNFα (0.03–10 ng mL^−1^) and IL1‐β (0.05–5 ng mL^−1^) treatments for different durations on disrupting endothelial cell function and the potential of grape seed extracts (2.5–10 μg mL^−1^), resveratrol (0.1–10 μm), resveratrol conjugates and quercetin (0.1–25 μm) treatments to overcome the deleterious effects of these aforementioned conditions on endothelial cell function using approaches targeted on specific endothelial cell function markers. According to our preliminary unpublished data, 10 ng mL^−1^ TNFα and 10 μm quercetin exerted the most potent disruptive and protective effects respectively. Nevertheless, glucose did not seem to disrupt endothelial cell function according to the results of the targeted experiments. Therefore, the concentration and incubation time of glucose used for metabolomics study were decided based on data obtained from previous studies where glucose treatments of HUVECs showed pro‐inflammatory effects.^[^ ^27^, ^28^, ^29^ ^]^

In the present study, we have shown that high‐glucose concentrations and treatment with the pro‐inflammatory cytokine TNFα alter endothelial cell metabolism leading to what would be regarded as an unhealthy metabolic phenotype, and show that this can be ameliorated by treatment with quercetin and its phase‐2 metabolites. In our study we have shown that high‐glucose concentrations, TNFα and quercetin treatments affected mainly HUVEC energy metabolism. This was obvious with the changes in intracellular inosine, adenosine, AMP, ADP, ATP, lactate, pyruvate, NAD^+^ and amino acids feeding Krebs cycle such as glutamate, aspartate and asparagine. High‐glucose stimulation increased intracellular lactate and glutamate concentrations compared to resting cells. Pre‐treatment with quercetin prior to high‐glucose stimulation was shown to suppress the increase in intracellular lactate concentrations. In parallel, increases in inosine and reductions in ATP concentrations were observed with quercetin pre‐treatment. All these changes are likely to be associated with the anti‐inflammatory properties of quercetin. Elevated lactate concentrations were observed with high‐glucose treatments since HUVECs preferably use glycolysis for energy production rather than the Krebs cycle and convert glucose to lactate even in the presence of oxygen.^[^ ^30^ ^]^ This phenomenon is also likely to explain the increase in glutamate concentration with high‐glucose treatment. Glutamate can be converted to the Krebs cycle intermediate α‐ketoglutarate which feeds into the Krebs cycle for auxiliary ATP production. Nevertheless, increased glucose load favors the use of glycolysis limiting the utilization of glutamate. Interestingly, quercetin treatment repressed lactate concentrations most likely by directing pyruvate flux into Krebs cycle.^[^ ^31^ ^]^ This notion is supported by the reduction in intracellular NAD^+^ concentration which indicated that NAD^+^ was being reduced into NADH in the Krebs cycle with quercetin stimulation. In the literature, there are several studies that reported pro‐atherogenic properties of lactate such as increased reactive oxygen species production (ROS) which in turn leads to NF‐кB activation in HUVECs^[^ ^32^ ^]^ and increased vascular endothelial cell growth factor (VEGF) and VEGF2 protein concentrations which in turn increases HUVEC migration and IL‐8 production.^[^ ^33^ ^]^ Therefore, the reduced lactate concentrations with quercetin treatments observed in our study are in line with its anti‐atherogenic activity. Beside its anti‐atherogenic activity, reduced lactate concentrations with quercetin treatment demonstrates also its anti‐carcinogenic effects since most cancer cells prefer to use glycolytic pathway extensively to produce energy rather than Krebs cycle due to Warburg effect.^[^ ^34^ ^]^

The other changes are observed mainly in purine metabolism as increases in anti‐inflammatory metabolites inosine and adenosine concentrations together with the reduction in inflammatory metabolites ATP and ADP. In normal circumstances molecules like ATP, ADP, AMP and NAD^+^ are intracellular metabolites. Identification of any of these metabolites in the culture medium may indicate leakage of intracellular metabolites into culture medium during quenching or extraction process.^[^ ^35^ ^]^ None of these metabolites were identified neither with ^1^H NMR nor MS methods employed in the present study confirming the quality of the protocol used. Nevertheless ATP can be released into extracellular environment during inflammation by particular inflammatory cells,^[^ ^36^ ^]^ and it was previously shown that TNFα induces ATP release by HUVECs.^[^ ^37^ ^]^ Although it has been observed that HUVECs release ATP into the extracellular medium during acute inflammation, we were not able to detect ATP in the extracellular medium. The reason is likely to be the different incubation times used in the studies. Extracellular ATP was shown to exert its pro‐inflammatory activities in various ways through plasma membrane purinergic receptors. These include enhancement of production of inflammatory mediators such as IL‐1, IL‐8, monocyte chemoattractant protein‐1, growth regulated oncogene α and also activation of cell adhesion molecules in endothelial cells.^[^ ^38^ ^]^ Therefore, reductions in intracellular ATP concentrations with quercetin treatments observed in the present study is important as this limits the amount of ATP that may leak into extracellular environment after an inflammatory stimulus. Similarly, ADP is another potent inflammatory metabolite. It can regulate platelet reactivity in vascular injury sites and show pro‐aggregatory activity.^[^ ^39^ ^]^ However, its subsequent hydrolysis to AMP and then to adenosine inhibits platelet aggregation.^[^ ^39^ ^]^ Therefore, reduction in ADP concentration with quercetin treatment is another anti‐inflammatory process observed in the present study.

On the other hand, adenosine is a potent anti‐inflammatory metabolite which acts through G‐protein‐coupled P1 purinergic receptor (A_2A/2B_AR). It has been reported to reduce NF‐кB activation in various cell types,^[^ ^40^ ^]^ reduce cell adhesion molecule expression in endothelial cells^[^ ^41^ ^]^ and increase basal endothelial barrier function.^[^ ^42^ ^]^ Beside its anti‐inflammatory effects, adenosine may possess vasodilatory effects. These effects can be exerted through ATP‐sensitive K^+^ (K_ATP_) channels.^[^ ^43^ ^]^ Several studies stated that K_ATP_ channels have the potential to function as metabolic sensors and provide cardioprotective regulations by coupling cellular metabolism to membrane excitability when the cells experience disturbances such as hyperglycemia.^[^ ^44^, ^45^ ^]^ For instance, adenosine was shown to act through A_2_ adenosine receptors and activate K_ATP_ channels.^[^ ^43^, ^46^ ^]^ Activated K_ATP_ channels allow K^+^ efflux hyperpolarizing the endothelial cells which leads to Ca^2+^ influx. Elevated intracellular Ca^2+^ concentrations were shown to up‐regulate calcium/calmodulin‐dependent protein kinase II (CaMKII) in endothelial cells.^[^ ^47^ ^]^ Subsequent phosphorylation of CAMKII leads to activation of endothelial nitric oxide synthase (eNOS) and protein kinase B (Akt) which may increase nitric oxide production that would improve endothelial cell function. Decreased intracellular ATP and increased intracellular ADP concentrations were also shown to activate K_ATP_ channels.^[^ ^48^ ^]^ Elevated intra‐ and extracellular adenosine and decreased intracellular ATP concentrations observed in this study after quercetin treatments bear the potential to activate K_ATP_ channels and exhibit vasodilatory effects. Inosine was another metabolite reported to reduce inflammatory cytokine production showing anti‐inflammatory effects both in vitro and in vivo studies.^[^ ^49^, ^50^ ^]^ Therefore, adenosine and inosine increases are also anti‐inflammatory processes observed in the present study.

**Figure 4:**
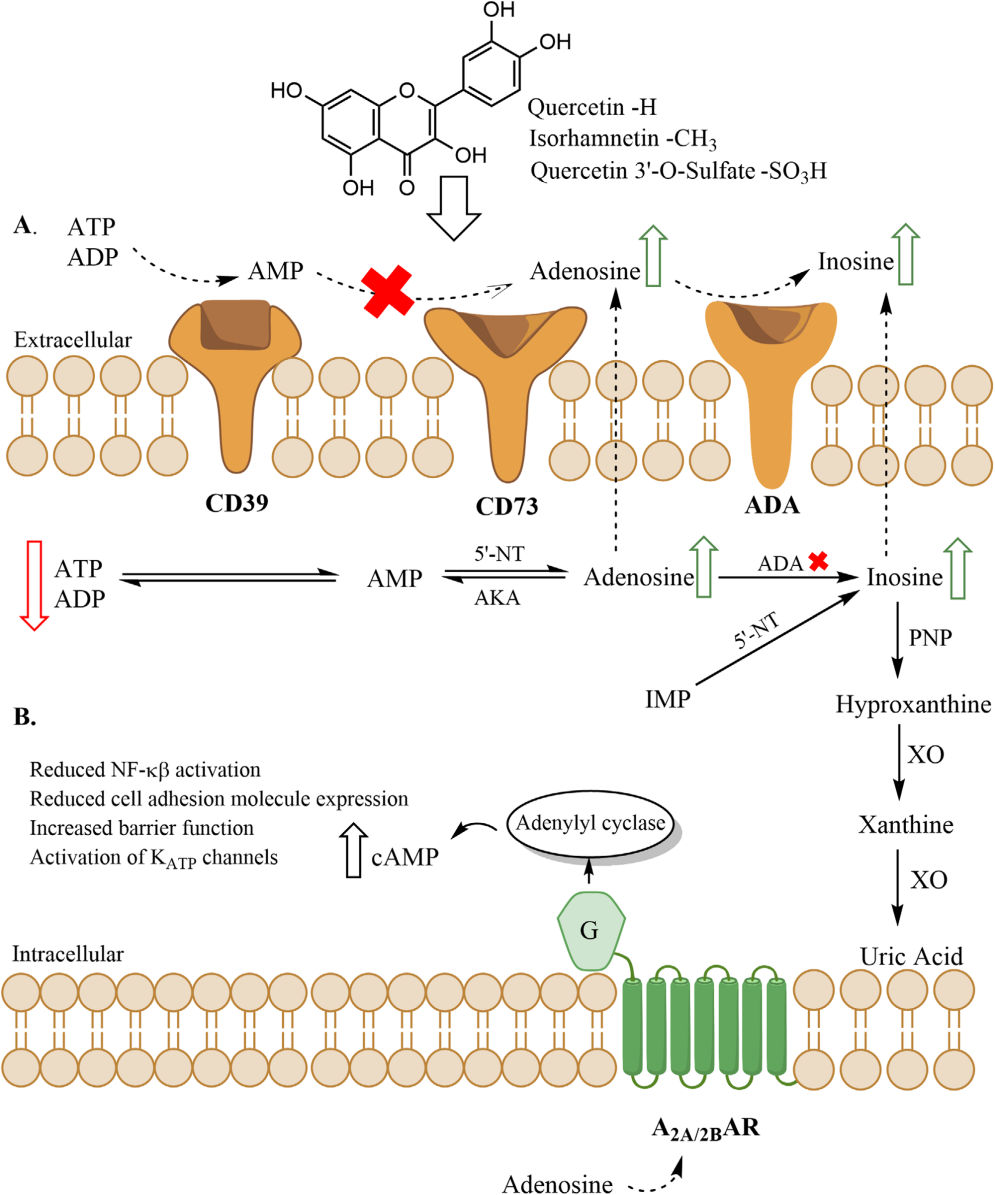
Purine metabolism pathway. A) The effects of quercetin and selected quercetin conjugates on purine metabolism enzymes. The red arrows indicate the metabolites whose concentrations were increased and the green arrows indicate the metabolites whose concentrations were decreased after quercetin treatments (AKA, adenosine kinase, IMP, inosine 5′‐monophosphate). Red crosses indicate enzyme inhibition by quercetin or its conjugates. B) Hypothetical activation of adenosine receptors by increased adenosine concentrations leading to various anti‐inflammatory responses.

Energy metabolites such as ATP, ADP and adenosine are actively involved in cellular signaling cascades, and several polyphenols have been shown to affect the turnover of these metabolites by regulating activities of the enzymes involved in energy metabolism.^[^ ^51^, ^52^ ^]^ After investigating metabolism of quercetin in HUVECs, we explored the effects of quercetin aglycone and its conjugates on the major purine metabolism enzymes which converts ATP into uric acid gradually (**Figure** **4**). In the human body, quercetin is extensively metabolized into its conjugates after its consumption.^[^ ^53^ ^]^ Nevertheless, the biological effects of quercetin conjugates may not be as consistent as quercetin aglycone,^[^ ^54^ ^]^ and there are several in vivo and in vitro studies arguing that quercetin conjugates, especially glucuronosyl‐conjugates, may be deconjugated at the sites of inflammation by the β‐glucuronidase activity yielding quercetin aglycone which may be responsible for the biological effects observed.^[^ ^55^, ^56^ ^]^ Accordingly, we chose to use quercetin aglycone to treat the cells in the metabolomics part of the study followed by our study on selected purine metabolism enzymes in which we also investigated the effects of quercetin conjugates.

Quercetin and its conjugates were shown to be strong inhibitors of rhCD39. However, quercetin and the conjugates which were identified in the cells and culture medium did not inhibit CD39 enzymatic activity in the intact HUVECs. This result is in line with the observation of decreased ATP and ADP levels after quercetin (20 h) treatments which is most likely due to increased expression of CD39 protein by HUVECs since a previous study showed that quercetin increased cellular expression of CD39.^[^ ^51^ ^]^

5′‐Nucleotidases can be found either membrane bound (ecto‐5′−‐nucleotidase/CD73) or in soluble form in the cells.^[^ ^57^ ^]^ However in the present study we have tested our polyphenols only on CD73 (ecto‐5′‐nucleotidase) activity since high cellular ATP concentrations deactivates soluble 5′‐nucleotidase function.^[^ ^58^ ^]^ The only flavonol that inhibited CD73 activity in intact HUVECs was quercetin, and the inhibition was only partial and requiring relatively high quercetin concentrations (50 μm). Nevertheless, the increased AMP concentration with quercetin treatment in the present study may be explained by a possible elevation in CD39 protein expression by HUVECs, the minor inhibition of CD73 with quercetin or both. Subsequent demonstration of ADA activity inhibition in HUVEC extracts by quercetin, isorhamnetin, and Q3′‐O‐S is of importance since we have shown in our study that quercetin aglycone was absorbed into HUVECs and metabolized into methylquercetin and Q3′‐O‐S after 2 h quercetin treatment. Initial studies indicated that ADA activity could be detected only intracellularly.^[^ ^59^ ^]^ However, more recent studies confirmed extracellular ADA activity^[^ ^60^ ^]^ which together with our data on ADA inhibition by quercetin aglycone and its conjugates explained increases in intra‐ and extracellular adenosine (112.5% and 286.2% of control levels, respectively) concentrations after quercetin treatments.

It was obvious that inhibition of bacterial PNP is affected by conjugation of quercetin with methyl or glucuronide groups as only quercetin and Q3′O‐S inhibited the enzyme. Since the competition of sulfate with P_i_ for the PNP active site was reported earlier,^[^ ^61^ ^]^ we have tested the effects of increased sulfate ion and P_i_ ion concentrations on bacterial PNP activity and their possible effects on Q3′‐O‐S. Neither sulfate nor phosphate ions affected bacterial PNP activity or the inhibitory activity of Q3′‐O‐S indicating that inhibitory activity of Q3′‐O‐S was not due solely to the conjugated sulfate group. In the light of no inhibition of PNP activity in HUVECs with quercetin treatments, the time‐dependent increase in both intra‐ and extracellular inosine concentrations is likely to be explained by a possible decrease in the levels of PNP protein with time after quercetin treatments that might have diminished the rate of inosine conversion to hypoxanthine.

There are several studies in the literature which assessed the effects of various polyphenols including quercetin and its conjugates on xanthine oxidase activity.^[^ ^62^, ^63^ ^]^ In our metabolomics study, quercetin treatment did not cause any alterations in xanthine concentrations indicating that xanthine oxidase activity might not be affected by quercetin treatments in HUVECs. Nevertheless, we could not confirm that since the enzyme activity in HUVEC extracts could not be detected with assay used in this study. Another recent study which also explored the effects of quercetin and its conjugates on xanthine oxidase, PNP, and ADA activities found similar results where they could not initially detect xanthine oxidase activity in human plasma and red blood cells but observed strong inhibition of bovine milk xanthine oxidase activity with quercetin and Q3′O‐S. In human plasma, they did not observe any inhibition of PNP activity with quercetin which is also in line with the findings in this study where no inhibition of PNP activity was observed in HUVEC extracts with quercetin.^[^ ^64^ ^]^ Nevertheless, they observed only a weak inhibition of ADA activity in human plasma with quercetin but quercetin and its conjugates were shown to be potent inhibitors of ADA activity in HUVEC extracts in the present study. They emphasized also the potential role of hemodynamic forces on HUVECs.^[^ ^64^ ^]^ Endothelial cells may be affected from hemodynamic forces they experience during blood flow. HUVECs are naturally exposed to laminar shear stress in the umbilical cord and the laminar shear stress is associated with athero‐protective properties.^[^ ^65^ ^]^ There are several studies comparing the effects of laminar shear stress and oscillatory shear stress on HUVECs.^[^ ^65^, ^66^ ^]^ However, Tumova and colleagues compared the expression of selected genes in HUVECs grown under static conditions and laminar shear stress. They revealed increased expression of eNOS, glucose transporter (GLUT)‐3, VEGF, and hemeoxygenase (HO‐2) mRNA whereas ATP binding cassette subfamily B member 1 (ABCB1), ATP binding cassette subfamily C member 2 (ABCC2), ATP binding cassette subfamily G member 2 (ABCG2), monocarboxylate transporter 1 (MCT1), MCT4, MCT5, 6‐phosphofructo‐2‐kinase/fructose‐2,6‐bisphosphatase 3 (PFKFB3), organic anion‐transporting polypeptide 4C1(OATP4C1) and GLUT1 mRNA expression levels were not affected by laminar shear stress.^[^ ^64^ ^]^ A potential increase in GLUT3 levels may be relevant to present study since such a change would allow more glucose in HUVECs further elevating gradual lactate concentrations. Nevertheless, the rate of glycolysis would not be affected as there were no changes expected in phosphofructokinase levels, which is the rate‐limiting enzyme in glycolysis. At the same time unaffected MCT levels would not affect lactate transport from the cells. Similarly, transport of hydrophobic molecules and organic anions appeared not be affected as mRNA expression of ABC transporter genes and OATP4C1 did not change respectively. Therefore, we may suggest the consideration of potential effects of shear stress on endothelial cells in future studies.

In conclusion, we have explored the HUVEC metabolite profile and identified and quantified 27 metabolites. Alterations in HUVEC metabolic profile were demonstrated in response to inflammatory conditions and quercetin was shown to ameliorate some of the glucose‐induced changes. The fate of the quercetin in HUVECs was determined revealing that quercetin enters the cells and gets metabolized into quercetin conjugates. Quercetin was shown to alter the balance of HUVEC metabolites towards a less inflamed phenotype, both alone and in the presence of pro‐inflammatory stimuli. These alterations were shown to be consistent with the inhibition of particular enzymes involved in purine metabolism by quercetin and its HUVEC metabolites.

## Conflict of Interest

The authors declare no conflict of interest.

## Author Contributions

B.O. and P.A.K. designed the research; B.O. undertook the experimental research under the supervision of P.A.K.; G.L.G. advised on metabolomics analysis; B.O. wrote the manuscript; all authors read and approved the final version of the manuscript.

## Supporting information

Supporting information

## Data Availability

The data that support the findings of this study are available from the corresponding author upon reasonable request.
