# Supplementary material for: Anti‐Inflammatory Effects of Quercetin on High‐Glucose and Pro‐Inflammatory Cytokine Challenged Vascular Endothelial Cell Metabolism
Source: Mol Nutr Food Res. 2021 Jan 22;65(6):2000777. doi: 10.1002/mnfr.202000777 (PMC8614122; doi:10.1002/mnfr.202000777)
Supplement: Supplementary file 1 — Supporting information [file MNFR-65-2000777-s001.docx]

Table 1: Gradient profile of mobile phase for LC-DAD and LC-MS analyses.

| Time (min) | A % | B % |
| --- | --- | --- |
| 0 | 83 | 17 |
| 2 | 83 | 17 |
| 7 | 75 | 25 |
| 15 | 65 | 35 |
| 20 | 50 | 50 |
| 25 | 0 | 100 |
| 30 | 0 | 100 |
| 35 | 83 | 17 |
| 50 | 83 | 17 |

Table 2: Intracellular metabolites detected in HUVECs. 27 metabolites were identified of which 17 were amino acids. m, multiplet; t, triplet, d, doublet;q, quartet, s, singlet

| **No** | **Metabolites** | **Abbreviation** | **^1^H NMR Signals** |
| --- | --- | --- | --- |
| **1** | Leucine | Leu | 0.97(t), 1.70(m), 3.74(m) |
| **2** | Isoleucine | Ile | 0.94(t), 1.00(d), 1.27(m), 1.46(m), 1.96(m). 3.66(d) |
| **3** | Valine | Val | 0.99(d). 1.08(d), 2.28(m), 3.62(d) |
| **4** | Threonine | Thr | 1.34 (d), 3.60(d), 4.24(m) |
| **5** | Alanine | Ala | 1.48(d), 3.78(q) |
| **6** | Lysine | Lys | 1.49(m), 1.74(m), 1.92(m), 3.03(t), 3.76(t) |
| **7** | Arginine | Arg | 1.67(m), 1.92(m), 3.25(t), 3.79(t) |
| **8** | Pyroglutamate | Pyro | 2.03(m), 2.46(m), 2.35(m), 4.18(m) |
| **9** | Glutamate | Glu | 2.04(m), 2.14(m), 2.34(m), 3.75(m) |
| **10** | Glutamine | Gln | 2.14(m), 2.45(m), 3.78(t) |
| **11** | Pyruvate | Pyr | 2.38(s) |
| **12** | Methionine | Met | 2.14(m), 2.65(t), 3.85(dd) |
| **13** | Aspartate | Asp | 2.66(dd), 2.80(dd), 3.89(dd) |
| **14** | Asparagine | Asn | 2.88(m), 2.96(m), 4.00(m) |
| **15** | Tyrosine | Tyr | 3.03(m), 3.18(m), 3.93(m), 6.89(m), 7.18(m) |
| **16** | Phenylalanine | Phe | 3.10(dd), 3.26(dd), 3.99(dd), 7.32(d), 7.42(dd) |
| **17** | Histidine | His | 3.13(dd), 3.25(dd), 7.10(s), 7.90(s) |
| **18** | Glycine | Gly | 3.54(s) |
| **19** | Lactate | Lac | 1.33(d), 4.12(q) |
| **20** | Acetate | - | 1.92 (s) |
| **21** | Choline | Cho | 3.12(s), 3.53(m), 4.05(m) |
| **22** | Glucose | Glc | 3.25(dd), 3.41(m), 3.49(m), 3.55(dd), 3.75(m), 3.82(m), 3.91(dd), 4.63(d), 5.24(d) |
| **23** | Inosine | Ino | 4.30(q), 4.44(q), 6.10(s), 8.25(s), 8.36(s), |
| **24** | Adenosine triphosphate | ATP | 6.16(d), 8.24(s), 8.53(s) |
| **25** | Nicotinamide adenine dinucleotide | NAD | 8.14 (s), 8.20(m), 8.41 (s), 8.51(s), 9.13(d), 9.33(s) |
| **26** | Adenosine diphosphate | ADP | 5.94(m), 8.29(s), 8.54(s) |
| **27** | Formate | - | 8.46 (s) |

Table 3: M-Z values for the quercetin and its conjugates detected in the culture medium samples confirming their identities.

| Retention Time (min) | M-H | M+H | Identity |
| --- | --- | --- | --- |
| 16.56 | 380.8 | 382.9 | Q 3’-O-S |
| 17.73 | 600.8 | - | Putative Quercetin Dimer |
| 19.95 | 300.9 | 302.9 | Quercetin |
| 21.36 | 628.8 | - | Putative Methylquercetin Dimer |
| 22.55 and 22.67 | 314.9 | 317.0 | Methylquercetin |
